# Intermarriage

**Published:** 1851-10

**Authors:** 


### BIBLIOGRAPHICAL.

*Intermarriage: or the mode in which, and the causes why, Beauty, Health, and Intellect result from certain Unions and Deformity—Disease and Insanity from others; demonstrated by Deliniations of the structure and forms, and descriptions of the functions and capacities which each parent, in every pair, bestows on children. In conformity with certain natural laws, and by an account of corresponding effects in the Breeding of Animals, with eight illustrative drawings.* By ALEXANDER WALKER. PHILADELPHIA: LINDSAY & BLAKISTON, 1851, 12 mo, pp. 384.

THE preliminary of this work is devoted to a very brief statement of a few anatomical and physiological facts, which are necessary as the ground work for the understanding of the proposed doctrine. It divides the human system into three classes of organs and functions, 1st. The *locomotive, vital* or *nutritive*, and the *mental* or *thinking*. The first is made to consist of bones, ligaments and muscles, by which all motion is produced. The second of lacteals, blood vessels and glands, which perform the functions of nutrition, secretion and excretion. The third of the special organs of sense, a brain which perceives, compares, reflects, &c., a cerebral or smaller brain, posterior to and below the former, which swells and gives action to the muscles, and this, power and motion, and from this latter source is derived sense, thought, motion and all connection with external objects.

Following this, is a tabular sheet, with a systematical arrangement of these various organs, with their respective offices, the knowledge of which, is indispensable to the full comprehension of the subject.

PART I.—Treats of the physiological conditions connected with, and terminating in love.

PART II.—Sexual relations arising from these conditions, and connected with a leading to intermarriage.

PART III.—Circumstances resulting from the preceding relation, and connected with, a production of progeny.

PART IV.—Newly discovered natural laws, regulating the resemblance of progeny to parents.

PART V.—Vague methods of regulating progeny, adopted in the breeding of domesticated animals.

Part VI.—Application of the natural laws, to the breeding of domesticated animals.

Part VII.—Vague methods affecting progeny, adopted among mankind.

Part VIII.—Choice in intermarriage, and prescribed by the natural laws.

Part I.—*Section* 1. Assigns the cause of the earlier puberty of women over men, to be in the proportionally larger vital or nutritive system. In earlier life, the three classes of organs are considered equal, the girl not distinguishable from the boy in these particulars, until a gradual change occurring at different ages, according to the various influences of ***climate, aliment, temperament,*** &c., produces puberty, and which the author affirms to be dependent upon the magnitude of what he terms the vital system, liable to modification from various external influences.

Temperature of climate modified, produces marked effects, but which is far excelled by peculiar kind of food, as very nutritious, stimulating meats, aromatics, the habitual use of coffee, wine, liquors, &c. These hasten this period, whilst farinaceous substances, roots and vegetable diet, with the habitual use of milk, cheese, &c. rather retard it. Assuming such to be facts, he asserts that the rich and inhabitants of towns, reach maturity sooner than the poor and the peasantry, also the moral condition, from the different influences dependent on aliment, bearing upon these two classes of people, produce a striking difference in the periods of puberty.

That the retardation of puberty, retards the development of intellectual powers, but preserves energy and freshness of sentiment, and by giving better development to the body, produces longer life, but if this state be prolonged after the ordinary period, woman appears to approximate to man in taste and some external characteristics.

The statistics of puberty in different parts of the world is highly interesting, which are as follows: In Europe, women reach puberty later in the north than in the south, in the most elevated northern regions it does not occur till after twenty years of age. In England at about fifteen in girls, and seventeen in boys. In many parts of France it commences generally at fourteen years of age, but in the southern departments, at thirteen. In Italy and Spain it takes place at twelve, and marriage often occurs at twelve. In Greece it is usual at ten. In Persia at nine or ten, which is also the usual age of this period in Arabia, Barbary, Egypt, Abyssinia, Senegal and various parts of Africa. Siberian women of the Mongolian race are marriageable at the age of thirteen, in a climate as cold as that of Sweden, whilst the Swedish female is scarcely so at fifteen or sixteen. But what is most remarkable by this statement is, that the Lamoides, inhabitants of the polar regions, are nubile at eleven, and frequently mothers at twelve.

We find that the precocious development of maturity before completion of growth, diminishes stature, depreciates beauty and intellect, and produces premature age.

In sections 2 and 3 is given the changes in the locomotive system, as well as in the vital, by the growth of the muscles, bones, and the general stature; thus accounting for the change of voice of lads and girls having reached pubescence; following, is a summary of physiological phenomena related in a very popular style, which cannot fail but to be instructve, and highly useful to the general reader.

The causes of many diseases and deformities affecting health, and the function of generation are here given. The striking peculiarities of sex are also shown to be dependent upon peculiar organic structure and habit. The want or interference with this structure, followed by certain consequences, detrimental to perfect development.

In sections 4, 5 and 6, much is said about love, affections, moral influences, &c., and physiology is taxed to its utmost to establish these as physiological results, and is treated in modest, pretty language, but is better calculated to please the young, than the old, and yet could hardly be dispensed with to complete the design of this work.

Part II—treats of several relations arising from mentioned conditions, and connected with, or leading to intermarriage, under the heads of *useful guidance, dangerous restraint, necessity of intermarriage.*

Part III.—Circumstances resulting from the preceding relations, and connected with, or production of progeny, under the head of *natural preferences of the various kinds of beauty, for the first time explained, state of marriage, forms and qualities propagated.*

Part IV—Newly discovered natural laws, regulating the resemblance of progeny to parents, under the head of *laws of resemblance, laws of selection,* &c. We deem it unnecessary to go farther, simply for the reason that in parts V, VI, VII, and VIII, much is said that is, to say the least, repugnant, if not unnecessary, to the class of readers that this work is intended to serve, thinking that the subject might have been so treated, as to have fitted more for social reflection, than for the farrier or breeder of stock. That abundant facts, more striking and acceptable, could be found to establish all points, if the author had confined himself to illustrations more human. The mixing up of "approximal diversities" with "dogs, pigs and horses," seems to us a strange mistake, in a work calculated for moral and intellectual improvement. We are lovers of comparative anatomy, but not of this stamp, and regret that we have aught to find fault with, connected with so much that is good and highly serviceable.

There are many so fastidious as to object to this treatise upon such delicate subjects, but we should think no one could attentively read it, and not receive new ideas of practical instruction, and much insight into the intricacies of human nature.

